# Autoimmune Hepatitis in Iran: What We Know, What We Don’t Know and Requirements for Better Management

**DOI:** 10.5812/hepatmon.840

**Published:** 2012-02-29

**Authors:** Mostafa Shafiei, Sayed Moayed Alavian

**Affiliations:** 1 Baqiyatallah Research Center for Gastroenterology and Liver diseases, Baqiyatallah University of Medical Sciences, Tehran, IR Iran

**Keywords:** Hepatitis, Autoimmune, Iran, Treatments

Autoimmune hepatitis (AIH) has been the subject of numerous studies, from the late 1940s when it first came to clinical attention, and various aspects of this disease have been analyzed throughout the world [1]. Although its clinical, laboratory and histological features, as well as demographical characteristics, have been extensively studied in many countries and in different ethnic groups, there have been no such statistics or analysis available for Iranian patients with AIH until now. The number of Iranian studies published so far about autoimmune hepatitis has been very limited. We only have two studies concerning the clinical characteristics of Iranian adult patients with AIH [2][3] and the information obtained can be summarized as follows: Iranian patients with AIH are typically women (male/female: 1:4), usually in their 30s and almost all of them have type 1 of the disease. We also have other Iranian studies regarding coeliac disease in patients with AIH [4][5], studies about cyclosporine therapy for AIH [6][7], surveys on the impact of immunosuppressive treatment on cirrhosis and liver fibrosis in AIH [8][9] and finally, some scattered studies related to the role of viruses and gene polymorphisms in AIH disease etiology [10][11][12][13]. However, there has not been any rigorous research conducted so far on the long-term outcomes of AIH in Iranian patients. In this issue of Hepatitis Monthly, Malekzadeh et al. analyzes both the clinical features and long term treatment outcomes of 102 Iranian patients diagnosed with AIH [14]. This article is both the first and the largest study detailing clinical aspects of AIH and long term outcomes based on data gathered from Iranian patients. According to the findings of this study, Iranian patients with AIH have several characteristics that are similar to other reported AIH series, these include; two age-peaks at presentation, clinical biochemical and histopathological features when AIH is first diagnosed, concurrent autoimmune diseases, and response rates to corticosteroids. We also now know that Iranian patients with AIH have an excellent 10-year survival rate of 96%, which is consistent with findings from previous studies carried out on other ethnic groups.

AIH is a rare disease and there are only a few credible data sources on the epidemiological aspects of the disease. For example, we know that the incidence of AIH in New-Zealand is 2 cases per 100,000 persons and its point prevalence is 24.5 cases per 100,000 persons [15]. The point prevalence of AIH in Alaska and Norway is 43 and 16.9 cases per 100,000 persons, respectively [16][17]. However, we don't have any similar data about the incidence or the prevalence of AIH among Iranians and thus it is impossible to compare Iranian data with other countries. Nevertheless, despite the fact that AIH accounts for only 2.6% of liver transplantation in the European Liver Transplant Registry [18] and 5.9% of liver transplantation in the National Institute of Health Liver Transplantation Database [19], a report by Saberifiroozi et al. showed that of the 480 patients who were over 14 years old and on the liver transplantation list from 1994 to 2004 in the Shiraz Center for Liver Transplantation, 64 cases (13%) were diagnosed with AIH [20] which is a much higher rate than what has been seen in the US and European countries.

In 1993, the International Autoimmune Hepatitis Group (IAIHG) codified diagnostic criteria for AIH and suggested a scoring system for its diagnosis [21]. These criteria were later revised by an expanded panel in 1999 [22]. The scoring system suggested by the IAIHG lists all the different presentations of this disease and the sum of the scores determines the accuracy of the diagnosis before and after treatment with glucocorticoids. Apart from the human leukocyte antigen (HLA) typing test and tests for detecting investigational autoantibodies including; the asialoglycoprotein receptor (ASGPR), anti actin, anti LC1 and anti SLA/LP, other tests related to the IAIHG scoring system are routinely available in Iranian laboratories. Although most of the studies on AIH carried out in Iran claim to use the IAIHG scoring system, in order to diagnose and categorize patients into definite and probable groups, none of them have any reports on HLA typing or frequency of investigational autoantibodies at presentation [2][4][23]. Another problem in Iran is the incorrect use of other diagnostic tests related to AIH. Common mistakes are the evaluation of positive/negative results without consulting the titration of serum autoantibody levels (ALKM-1, ASMA, ANA and AMA) and the incorrect interpretations of these tests.

According to the latest guidelines; titres equal to or more than 1:40 for all the autoantibodies in adults, and in children titres equal to or more than 1:20 for ANA and ASMA and the titres equal to or more than 1:10 for ALKM-1, are considered to be positive and have scoring values [24][25]. However, many Iranian medical centers only consider the test positive if the titres are equal to or more than 1:80.

Despite the fact that almost all of the credible references and guidelines recommend a liver biopsy, before the start of treatment for patients who are suspected to have AIH [24][25], we still see in Iran, patients that didn't have any contra-indication to undergoing liver biopsy but treated without it.

Consequently this leads to improper treatment of patients with glucocorticoids. For example up to 20% of patients with proven fatty liver disease based on their liver histology, had the criteria for a definite or probable diagnosis of AIH before undergoing a liver biopsy [26]. Furthermore, according to the revised criteria for AIH [22], the necessity for the disease to be present for six months to establish its chronicity has been deleted, nevertheless, some Iranian physicians still do not start treatment before establishing chronicity.

The efficacy of long-term monotherapy with a high dose of prednisone (USA), or prednisolone (European countries and also Iran), or in lower doses and in combination with azathioprine, was proven to be effective in the 1960s and since then this has been the standard treatment for AIH [27]. A common mistake in therapy of AIH among Iranian physicians is starting the treatment with non-efficient doses of glucocorticoids. In most references it has been emphasized that treatment should start with the maximum recommended dose of prednisolone (30 mg/day) in combination with 1-2 mg/kg/day of azathioprine [24][25], as doses less than this may lead to a delay in achieving remission and consequently postpone the achievement of treatment endpoint criteria.

Other issues which should be taken into consideration are the absolute and relative indications for treatment [24]. It is also very important to ensure that there are no contraindications for treatment such as; severe cytopenia, morbid obesity or severe osteoporosis [24]. Up until the present time, there have been many debates on the necessity of treatment for patients with relative indications for treatment. Nevertheless, in recent years these discussions have tended to emphasize the importance of treatment of asymptomatic patients or patients with a mild disease [28]. In earlier studies in the Mayo Clinic and numerous latter studies, biochemical remission of AIH had been defined as a decrease in AST serum levels to less than half of normal. Up to 80% of patients can achieve these criteria with prednisolone-based treatments [29]. This issue could have been confusing before, as IAIHG had used two different terms for remission: one was a decrease in AST serum levels to less than half of the upper normal limit and the other was full normalization of AST [25]. Indeed, this issue has now been resolved and almost all of the current credible guidelines state that complete normalization of serum transaminases must be the final treatment goal [24]. The importance of this issue is that even mild, but consistent elevations in serum transaminases, can be associated with; persistent hepatitis [30], relapse after treatment withdrawal [31], progression to cirrhosis [32], and poor prognosis [33]. Therefore a treatment endpoint which was considered to be a decrease in AST serum levels to less than half of normal, should be changed to full normalization [25].

There are still many important questions to be raised concerning practice details in current standard treatments. Besides these, other new and promising treatments are awaiting official comparisons with current standard regimes. Cyclosporine is one of the drugs which has proved its efficacy and safety in the treatment of AIH in many studies [34][35] including a survey by Malekzadeh et al. from Iran [7]. Unfortunately, there have been no reports on other alternative treatments for AIH in Iran. Among other new possible treatments are; budesonide and mycophenolate mofetil, the efficacy of the former has been recently proved in a big multicentral cohort study [36], and the later seems to be a good alternative for azathioprine [37]. Ursodeoxycholic acid which is usually used as an adjuvant therapy in the treatment of AIH in Iran, is only efficient for AIH patients with concurrent overlap syndromes such as primary biliary cirrhosis (PBC) and primary sclerosing cholangitis (PSC), but its efficacy has not been proven in pure AIH cases [38].

Other alternative strategies in cases of treatment failure are the administration of; tacrolimus, methotrexate, cyclophosfamide and biologic treatments such as; etanercept, rituximab and infliximab [38]. There have only been a few cases of using each of these strategies and in most cases the result were encouraging, but not reassuring enough to reach a consequence. At the end, it should be emphasized that AIH is a disease that needs to be managed under the supervision of a team of specialists, including at least one hepatologist or a gastroenterologist with an interest in liver disease, a specialist liver histopathologist, as well as a laboratory that can measure all serum autoantibodies with accurate titres. Regular meetings held to discuss complicated cases are also very helpful and can prevent physicians from mismanagement.

In conclusion, it seems that we are just beginning to understand the condition of AIH in Iran, as the number of studies that have been conducted on AIH in Iran scarcely exceeds the number of fingers on both hands. This issue indicates the importance of designing multiple new studies on different aspects of AIH in Iran. Clinical, laboratory and histological features of Iranian patients with AIH have still not been properly identified, so the first step required is that new studies must focus on these unknown aspects of the disease and especially on the differences among Iranian patients and other ethnic groups. Collaboration of major herpetology research centers in Iran in a big multicenter cohort study would be a crucially important combined project; this would enable an accurate estimation of the epidemiological characteristics of AIH in Iran and also the long-term outcomes of patients in various Iranian provinces.
